# The Aryl Hydrocarbon Receptor Governs Epithelial Cell Invasion during Oropharyngeal Candidiasis

**DOI:** 10.1128/mBio.00025-17

**Published:** 2017-03-21

**Authors:** Norma V. Solis, Marc Swidergall, Vincent M. Bruno, Sarah L. Gaffen, Scott G. Filler

**Affiliations:** a Division of Infectious Diseases, Department of Medicine, Los Angeles Biomedical Research Institute at Harbor-UCLA Medical Center, Torrance, California, USA; b Institute for Genome Sciences and Department of Microbiology and Immunology, University of Maryland School of Medicine, Baltimore, Maryland, USA; c Division of Rheumatology and Clinical Immunology, University of Pittsburgh, Pittsburgh, Pennsylvania, USA; d Department of Medicine, David Geffen School of Medicine at UCLA, Los Angeles, California, USA; Duke University Medical Center

**Keywords:** *Candida albicans*, aryl hydrocarbon receptor, epithelial cells, host cell invasion, interferon-gamma

## Abstract

Oropharyngeal candidiasis (OPC), caused predominantly by *Candida albicans*, is a prevalent infection in patients with advanced AIDS, defects in Th17 immunity, and head and neck cancer. A characteristic feature of OPC is fungal invasion of the oral epithelial cells. One mechanism by which *C. albicans* hyphae can invade oral epithelial cells is by expressing the Als3 and Ssa1 invasins that interact with the epidermal growth factor receptor (EGFR) on epithelial cells and stimulate endocytosis of the organism. However, the signaling pathways that function downstream of EGFR and mediate *C. albicans* endocytosis are poorly defined. Here, we report that *C. albicans* infection activates the aryl hydrocarbon receptor (AhR), leading to activation of Src family kinases (SFKs), which in turn phosphorylate EGFR and induce endocytosis of the fungus. Furthermore, treatment of oral epithelial cells with interferon gamma inhibits fungal endocytosis by inducing the synthesis of kynurenines, which cause prolonged activation of AhR and SFKs, thereby interfering with *C. albicans*-induced EGFR signaling. Treatment of both immunosuppressed and immunocompetent mice with an AhR inhibitor decreases phosphorylation of SFKs and EGFR in the oral mucosa, reduces fungal invasion, and lessens the severity of OPC. Thus, our data indicate that AhR plays a central role in governing the pathogenic interactions of *C. albicans* with oral epithelial cells during OPC and suggest that this receptor is a potential therapeutic target.

## IMPORTANCE

OPC is caused predominantly by the fungus *C. albicans*, which can invade the oral epithelium by several mechanisms. One of these mechanisms is induced endocytosis, which is stimulated when fungal invasins bind to epithelial cell receptors such as EGFR. Receptor binding causes rearrangement of epithelial cell microfilaments, leading to the formation of pseudopods that engulf the fungus and pull it into the epithelial cell. We discovered AhR acts via SFKs to phosphorylate EGFR and induce the endocytosis of *C. albicans*. Our finding that a small molecule inhibitor of AhR ameliorates OPC in mice suggests that a strategy of targeting host cell signaling pathways that govern epithelial cell endocytosis of *C. albicans* holds promise as a new approach to preventing or treating OPC.

## INTRODUCTION

Oropharyngeal candidiasis (OPC) is one of the most common opportunistic infections in HIV-infected individuals, occurring in up to 90% of those with advanced immune suppression (1, 2). The prevalence of OPC and esophageal candidiasis remains high in patients newly diagnosed with HIV, especially in Asia, Africa, and Latin America (3–6). *Candida albicans* causes at least 80% of cases of OPC in patients with HIV/AIDS (7, 8) and is also the most common cause of OPC in patients with Sjogren’s syndrome, diabetes mellitus, and cancer of the head and neck (9–11). The predominance of *C. albicans* as the cause of OPC suggests that this organism possesses unique characteristics that enable it to colonize the oropharynx and, when host defenses are impaired, cause OPC.

A characteristic finding during OPC is invasion of the superficial epithelium (12). Indeed, transmission electron microscopy studies of biopsy specimens from patients with OPC demonstrate organisms within the oral epithelial cells (13, 14). Candidal invasion of epithelial cells is a continuous process during OPC, occurring both when a focus of infection is initiated and as the lesion progressively expands. *C. albicans* can invade epithelial cells by two different mechanisms: active penetration and induced endocytosis (15–19). The latter process occurs when the *C. albicans* Als3 and Ssa1 invasin proteins bind to epithelial cell E-cadherin and a heterodimer consisting of the epidermal growth factor receptor (EGFR) and HER2. Binding to these receptors triggers rearrangement of epithelial cell microfilaments, leading to the formation of pseudopods that surround the organism and pull it into the epithelial cell (20–22).

As a prototypic Th1 cytokine, interferon gamma (IFN-γ) has been used as adjunctive therapy for patients with both hematogenously disseminated candidiasis and multidrug-resistant OPC (23, 24). When administered prophylactically to patients with advanced HIV infection, IFN-γ appears to reduce the frequency of OPC (25). The salutary effects of IFN-γ on the host’s defense against *C. albicans* infection have been thought to be due to enhanced antigen presentation and phagocyte activity (26). However, IFN-γ also has effects on nonmyeloid cells. Previously, we found that treatment with IFN-γ protects endothelial cells from *C. albicans* infection *in vitro* by inhibiting endothelial cell endocytosis of the organism (27). In the present study, we investigated the capacity of IFN-γ to protect oral epithelial cells from invasion by *C. albicans*. We found that 24 h of exposure of oral epithelial cells to IFN-γ activates indoleamine 2,3-deoxygenase (IDO), leading to the synthesis of kynurenines, which activate the aryl hydrocarbon receptor (AhR) and Src family kinases (SFKs). Prolonged activation of SFKs inhibits the phosphorylation of EGFR and reduces endocytosis of *C. albicans*. Pharmacological inhibition of the AhR inhibits SFK activation and endocytosis *in vitro* and reduces the severity of OPC in mice, indicating that this cytoplasmic receptor plays a vital role in the endocytosis of *C. albicans*, both *in vitro* and *in vivo*.

## RESULTS

### IFN-γ treatment inhibits endocytosis of *C. albicans* by oral epithelial cells.

**FIG 1:**
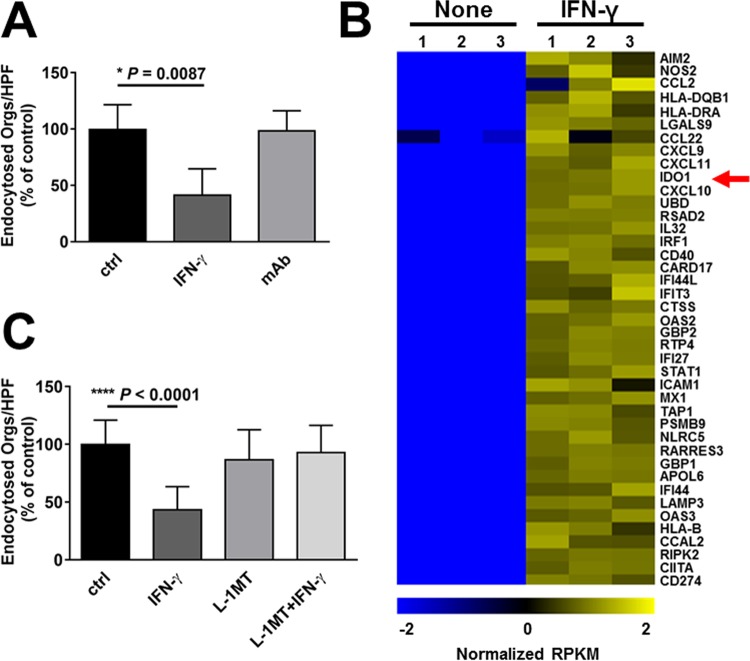
Interferon gamma (IFN-γ) inhibits endocytosis of *C. albicans* by oral epithelial cells. The OKF6/TERT-2 oral epithelial cell line was incubated with IFN-γ for 24 h in the absence and presence of an anti-IFN-γ monoclonal antibody (mAb). The cells were infected with *C. albicans* SC5314 for 2.5 h, after which the number of endocytosed organisms was determined using a differential fluorescence assay. Results are means ± standard deviations (SD) from three experiments, each performed in triplicate. Orgs/HPF, organisms per high-power field; ctrl, control. Statistical significance was determined using the unpaired Student’s *t* test (*P* ≤ 0.05). (B) RNA-seq analysis of the effects of IFN-γ on the transcriptional response of oral epithelial cells to *C. albicans*. OKF6/TERT-2 epithelial cells were incubated in the presence or absence of IFN-γ for 24 h and then infected with *C. albicans* for 5 h. RNA was extracted and analyzed by RNA-seq. The heat map shows normalized, log-transformed RPKM values of the top 40 IFN-γ-responsive genes. The red arrow indicates the IDO1 gene. (C) Effects of IDO inhibition with l-1-methyl-tryptophan (l-1MT) on OKF6/TERT-2 oral epithelial cell endocytosis of *C. albicans*. Results are means ± SD from three experiments, each performed in triplicate. Statistical significance was determined using the unpaired Student’s *t* test (*P* ≤ 0.05).

To investigate the effects of IFN-γ on the endocytosis of *C. albicans*, the OKF6/TERT-2 oral epithelial cell line (28) was incubated with either IFN-γ or medium alone for 24 h and then infected with *C. albicans* strain SC5314. The number of organisms endocytosed by the oral epithelial cells was measured by our standard differential fluorescence assay, in which endocytosed/internalized organisms fluoresced red, whereas nonendocytosed organisms fluoresced both red and green (20, 21, 29). We found that incubation of epithelial cells with IFN-γ reduced the endocytosis of *C. albicans* by approximately 60% (Fig. 1A). The inhibitory effect of IFN-γ was reversed by a monoclonal antibody that blocked the epithelial cell IFN-γ receptor.

To determine if IFN-γ influenced epithelial cell invasion via active penetration, we treated the epithelial cells with this cytokine for 24 and then fixed them with paraformaldehyde. After rinsing the cells extensively, we infected them with live *C. albicans* cells in the presence of IFN-γ. Although we detected active penetration of *C. albicans* into the fixed cells, this process was not affected by IFN-γ (see Fig. S1A in the supplemental material). Furthermore, IFN-γ had no detectable effect on *C. albicans* hyphal formation (Fig. S1B and C) or adherence to the epithelial cells (9.9 ± 3.7 cell-associated organisms per high-power field for control epithelial cells versus 10.3 ± 3.6 for IFN-γ-treated cells; *n =* 18, *P* = 0.73). Collectively, these data indicate that IFN-γ inhibits invasion of *C. albicans* by reducing its endocytosis by oral epithelial cells.

10.1128/mBio.00025-17.1 FIG S1  IFN-γ has no effect on active penetration into epithelial cells or hyphal growth. (A) OKF6/TERT-2 oral epithelial cells were incubated with IFN-γ for 24 h, fixed, and infected for 2.5 h with *C. albicans* cells in the presence of IFN-γ, after which the number of internalized organisms was determined by a differential fluorescence assay. Results are means ± SD from 3 experiments, each performed in triplicate. (B) Microscopic images of *C. albicans* cells after a 2.5-h incubation with oral epithelial cells that had been exposed to the indicated conditions for 24 h. The fungal cells were stained with an anti-*Candida* antiserum conjugated with Alexa Fluor 488. (C) Hyphal length after incubation for 2.5 h on oral epithelial cells that had been exposed to the indicated conditions for 24 h. Results are means ± SD from 50 organisms. Statistical significance was determined using the unpaired Student’s *t* test (*P* ≤ 0.05). ctrl, control; NS, not significant. Download FIG S1, PDF file, 0.2 MB. Copyright © 2017 Solis et al. 2017 Solis et al. This content is distributed under the terms of the Creative Commons Attribution 4.0 International license.

### IFN-γ upregulates its canonical targets in oral epithelial cells *in vitro*.

To gain more comprehensive insight into how IFN-γ decreases the endocytosis of *C. albicans*, we used transcriptome sequencing (RNA-seq) to analyze the transcriptional response of oral epithelial cells that were treated with IFN-γ and then infected with *C. albicans*. As expected, exposure to this cytokine resulted in upregulation of multiple IFN-γ target genes (Fig. 1B; see Table S1 in the supplemental material). Gene Ontology (GO) term analysis indicated that many of the upregulated genes were involved in the response to interferons (see Table S2 in the supplemental material). In contrast, treatment with IFN-γ did not significantly affect the mRNA levels of the EGFR, ERBB2 (HER2), or CDH1 (E-cadherin) genes that encode the epithelial cell receptors for *C. albicans* (Table S1).

10.1128/mBio.00025-17.8 TABLE S1  List of epithelial cell genes whose gene expression was altered by IFN-γ treatment. Download TABLE S1, XLS file, 0.4 MB. Copyright © 2017 Solis et al. 2017 Solis et al. This content is distributed under the terms of the Creative Commons Attribution 4.0 International license.

10.1128/mBio.00025-17.9 TABLE S2  GO term analysis of genes whose expression was up- or downregulated by IFN-γ treatment. Download TABLE S2, XLSX file, 0.1 MB. Copyright © 2017 Solis et al. 2017 Solis et al. This content is distributed under the terms of the Creative Commons Attribution 4.0 International license.

Among the known IFN-γ-responsive genes, the IDO1 gene was one of the genes most highly upregulated by IFN-γ treatment (Fig. 1B, red arrow). By real-time PCR, we verified that IFN-γ induced almost a 100-fold increase in IDO1 gene transcript levels in the oral epithelial cells (see Fig. S2 in the supplemental material), similar to what has been reported by others (30). To determine if IDO played a role in IFN-γ-mediated inhibition of endocytosis, epithelial cells were treated with IFN-γ in the presence of the IDO inhibitor l-1-methyl-tryptophan (l-1MT). We found that although l-1MT had no effect on the endocytosis of *C. albicans* by control epithelial cells, it completely reversed the inhibitory effects of IFN-γ (Fig. 1C), indicating that IFN-γ inhibits endocytosis by stimulating IDO activity.

10.1128/mBio.00025-17.2 FIG S2  Effect of IFN-γ on epithelial cell IDO1 mRNA expression, as measured by real-time PCR. Results are means ± SD from 2 experiments, each performed in triplicate. Statistical significance was determined using the unpaired Student’s *t* test (*P* ≤ 0.05). ctrl, control. Download FIG S2, PDF file, 0.3 MB. Copyright © 2017 Solis et al. 2017 Solis et al. This content is distributed under the terms of the Creative Commons Attribution 4.0 International license.

### IFN-γ inhibition of endocytosis is mediated by kynurenine.

**FIG 2:**
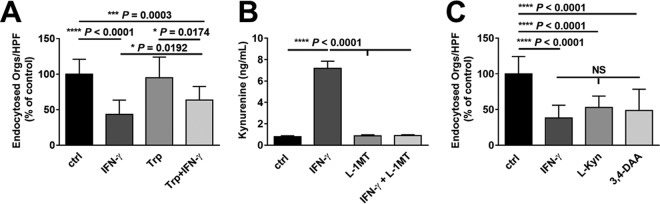
The tryptophan metabolite kynurenine inhibits the endocytosis of *C. albicans*. (A) Effects of IFN-γ and the indicated compounds on epithelial cell endocytosis of *C. albicans*. OKF6/TERT-2 oral epithelial cells were incubated for 24 h with tryptophan (Trp), either alone or in combination with IFN-γ, and then infected with *C. albicans* for 2.5 h. Results are means ± SD from three experiments, each performed in triplicate. (B) Kynurenine production by epithelial cells after incubation with the indicated compounds for 24 h. Results are means ± SD from three experiments. (C) Effects of 24 h of exposure to l-kynurenine (L-Kyn) or the kynurenine analog *N*-(3,4-dimethoxycinnamoyl)-anthranilic acid (3,4-DAA) on epithelial cell endocytosis of *C. albicans*. Results are means ± SD from three experiments, each performed in triplicate. Statistical significance was determined using the unpaired Student’s *t* test (*P* ≤ 0.05). NS, not significant; ctrl, control.

As the rate-limiting enzyme of tryptophan catabolism by the kynurenine pathway, IDO both degrades tryptophan and initiates the production of kynurenines (31). To determine if inhibition of endocytosis by IFN-γ was mediated by tryptophan depletion, we incubated epithelial cells with IFN-γ in the presence of exogenous l-tryptophan. Addition of l-tryptophan caused a small, but statistically significant reduction in IFN-γ-mediated inhibition of endocytosis (Fig. 2A). Next, we investigated whether the effect of IFN-γ on endocytosis was due to the enhanced production of kynurenine. First, we verified that treatment of oral epithelial cells with IFN-γ stimulated the release of l-kynurenine and that this process was blocked by the IDO inhibitor L-1MT (Fig. 2B). Next, we incubated oral epithelial cells for 24 h with either exogenous l-kynurenine or *N*-(3,4-dimethoxycinnamoyl)-anthranilic acid (3,4-DAA), the stable analog of a kynurenine metabolite (32). Both l-kynurenine and 3,4-DAA inhibited endocytosis of *C. albicans* similarly to IFN-γ (Fig. 2C). Collectively, these results support the model that IFN-γ stimulates IDO activity, leading to depletion of tryptophan and enhanced production of kynurenines and their metabolites, which inhibit the endocytosis of *C. albicans*.

### AhR activation of SFKs is required for maximal endocytosis of *C. albicans*.

**FIG 3:**
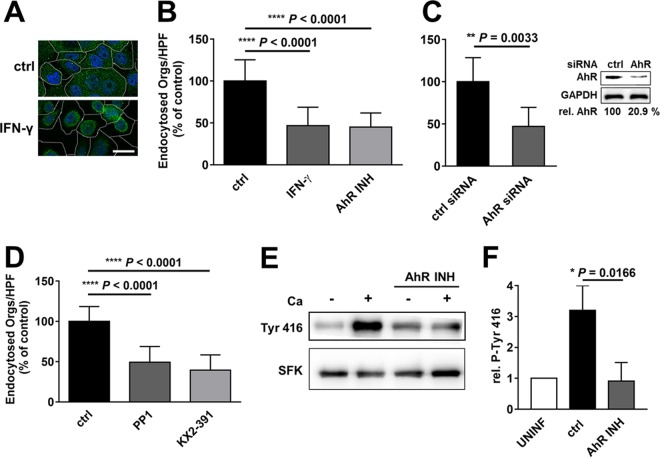
IFN-γ activates the aryl hydrocarbon receptor (AhR) and Src family kinases (SFKs), which govern the endocytosis of *C. albicans*. (A) Confocal micrographs of OKF6/TERT-2 oral epithelial cells incubated in the presence and absence of IFN-γ for 24 h. The cells were stained for AhR (green), and the nuclei were stained with DAPI (blue). The perimeters of the cells were determined by differential interference contrast and are indicated by the dashed lines. Scale bar, 20 µm. (B) Endocytosis of *C. albicans* by oral epithelial cells treated with IFN-γ for 24 h or the AhR inhibitor for 1 h. (C) Endocytosis of *C. albicans* by oral epithelial cells transfected with either control siRNA or AhR siRNA. The inset is a representative immunoblot showing knockdown of AhR. (D) Effects of 1 h of exposure to the indicated SFK inhibitors on the endocytosis of *C. albicans*. All endocytosis data are means ± SD from three experiments, each performed in triplicate. (E and F) Effects of *C. albicans* and the AhR inhibitor on SFK phosphorylation. OKF6/TERT-2 cells were pretreated for 1 h with the indicated inhibitor and then infected with *C. albicans* for 1 h, after which the extent of SFK phosphorylation was determined by immunoblotting. (E) Representative immunoblot. (F) Densitometric analysis of 3 immunoblots such as the one shown in panel E. Results are means ± SD from 3 experiments. Statistical significance was determined using the unpaired Student’s *t* test (*P* ≤ 0.05). ctrl, control; INH, inhibitor; Ca, *C. albicans*; UNINF, uninfected.

Kynurenines are endogenous ligands for AhR, which is located in the cytoplasm and forms a complex with SFKs (33, 34). When a ligand binds to AhR, the receptor translocates to the nucleus, while SFKs dissociate from the complex and become active, phosphorylating numerous substrates, including EGFR (35, 36). Using indirect immunofluorescence and confocal microscopy, we determined that treatment with IFN-γ caused AhR to translocate from the cytoplasm to the nucleus (Fig. 3A). Treatment with l-kynurenine also induced translocation of AhR (see Fig. S3 in the supplemental material), indicating that both IFN-γ and l-kynurenine activate AhR in oral epithelial cells.

10.1128/mBio.00025-17.3 FIG S3  l-Kynurenine activates the aryl hydrocarbon receptor (AhR) in oral epithelial cells. Shown are confocal micrographs of OKF6/TERT-2 oral epithelial cells incubated in the presence and absence of l-kynurenine for 24 h. The cells were stained for AhR (green), and the nuclei were stained with DAPI (blue). The perimeters of the cells were determined by differential interference contrast and are indicated by the dashed lines. Scale bar, 20 µm. ctrl, control. Download FIG S3, PDF file, 0.1 MB. Copyright © 2017 Solis et al. 2017 Solis et al. This content is distributed under the terms of the Creative Commons Attribution 4.0 International license.

To investigate whether AhR influences the endocytosis of *C. albicans* by oral epithelial cells, we incubated the cells for 1 h with the AhR inhibitor CH-223191 (37) prior to infection. This inhibitor reduced the endocytosis of *C. albicans* by the same extent as IFN-γ (Fig. 3B). Knockdown of AhR with small interfering RNA (siRNA) also significantly decreased *C. albicans* endocytosis (Fig. 3C). Therefore, AhR function is necessary for maximal endocytosis of the fungus.

Activation of AhR leads to derepression of SFKs, which undergo autophosphorylation and in turn phosphorylate and activate EGFR (35, 36). To determine whether SFKs govern epithelial cell endocytosis of *C. albicans*, we tested two structurally distinct SFK inhibitors, PP1 and KX2-391. Both inhibitors significantly reduced the endocytosis of the fungus (Fig. 3D). By immunoblotting with a phosphospecific antibody, we also determined that *C. albicans* infection of oral epithelial cells induced the tyrosine phosphorylation of SFKs (Fig. 3E and F). This phosphorylation was blocked when epithelial cells were incubated with the AhR inhibitor, indicating that AhR activation is required for SFK activity, which in turn is necessary for maximal epithelial cell endocytosis of *C. albicans*.

### IFN-γ, AhR, and SFKs govern endocytosis via phosphorylation of EGFR.

Our next objective was to investigate the relationship between IFN-γ and the epithelial cell receptors for *C. albicans*. One potential explanation for the inhibitory effects of IFN-γ on the endocytosis of *C. albicans* is that the cytokine downregulates the expression of one or more epithelial cell receptors for *C. albicans*. However, by real-time PCR, we verified our RNA-seq findings that IFN-γ did not change the mRNA levels of the genes encoding E-cadherin, EGFR, or HER2 (see Fig. S4A in the supplemental material). Furthermore, flow cytometric analysis indicated that IFN-γ treatment did not reduce the surface expression of these receptors (Fig. S4B). Therefore, IFN-γ must inhibit endocytosis by acting on another step in the endocytosis signaling pathway.

10.1128/mBio.00025-17.4 FIG S4  IFN-γ treatment has no effect on the expression of host cell receptors for *C. albicans*. (A) mRNA levels of the indicated epithelial cell receptors after 24 h of IFN-γ treatment. Results are means ± SD from two independent experiments performed in triplicate. (B) Effects of IFN-γ on the expression of E-cadherin, EGFR, and HER2 on the surface of epithelial cells as determined by flow cytometry. Control cells (stained with a control monoclonal antibody [MAb]) are shown in light gray, untreated cells (stained with the specific MAb) are shown in dark gray, and the IFN-γ-treated cells (stained with the specific MAb) are shown in red. Download FIG S4, PDF file, 0.3 MB. Copyright © 2017 Solis et al. 2017 Solis et al. This content is distributed under the terms of the Creative Commons Attribution 4.0 International license.

**FIG 4:**
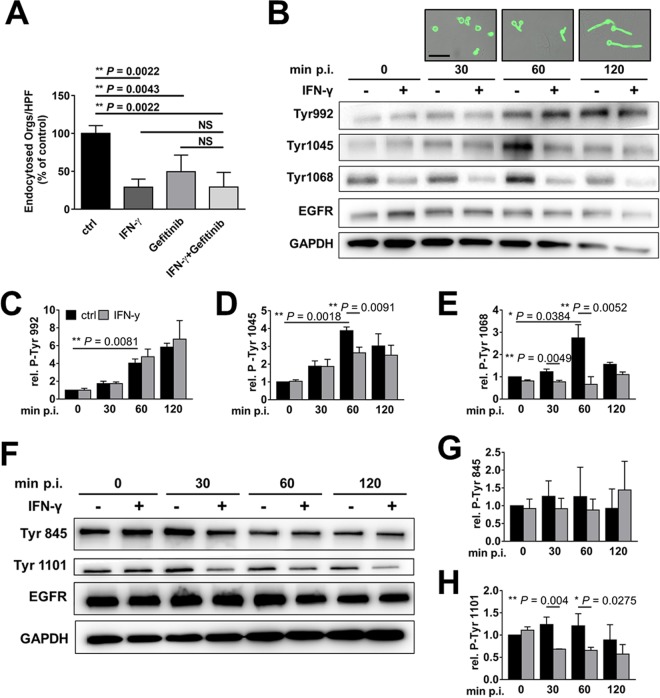
IFN-γ inhibits EGFR phosphorylation. (A) Effects of IFN-γ (24 h) and/or the EGFR inhibitor gefitinib (1 h) on the endocytosis of *C. albicans* by OKF6/TERT-2 oral epithelial cells. Results are the means ± SD from three experiments, each performed in triplicate. (B to E) Effects of IFN-γ on *C. albicans*-induced autophosphorylation of the indicated tyrosine residues of EGFR. The oral epithelial cells were incubated in the presence and absence of IFN-γ for 24 and then infected with *C. albicans* for the indicated time points. The phosphorylation of the specific EGFR tyrosine residues was determined by immunoblotting with specific monoclonal antibodies. (B) Representative immunoblots. The images above the blot show the *C. albicans* morphology at the indicated time points. Scale bar, 20 µm. (C to E) Densitometric analysis of the immunoblots. Results are means ± SD from 3 experiments. (F to H) Effects of IFN-γ on SFK-dependent phosphorylation of the indicated tyrosine residues of EGFR. (F) Representative immunoblots. (G and H) Densitometric analysis of the immunoblots. Results are means ± SD from 3 experiments. Statistical significance was determined using the unpaired Student’s *t* test (*P* ≤ 0.05). NS, not significant; ctrl, control; p.i., postinfection.

To investigate whether IFN-γ influences signaling through EGFR, we analyzed the effects of IFN-γ and the EGFR inhibitor gefitinib on endocytosis. Treatment of epithelial cells with either IFN-γ or gefitinib alone significantly reduced the endocytosis of *C. albicans* (Fig. 4A). Moreover, the inhibitory effect of combined treatment with both IFN-γ and gefitinib was similar to that of IFN-γ alone, suggesting that IFN-γ and gefitinib reduce endocytosis by inhibiting the same pathway.

EGFR is a receptor tyrosine kinase that, when activated, is autophosphorylated on multiple tyrosine residues, including Y992, Y1045, and Y1068 (38). To determine if IFN-γ alters *C. albicans-*induced autophosphorylation of EGFR, we treated oral epithelial cells with the cytokine and infected them with yeast-phase *C. albicans*. We observed that at 60 min postinfection, the organisms began to germinate, forming nascent germ tubes (Fig. 4B). By 120 min, these hyphae had grown considerably in length. At each time point, we lysed the cells and analyzed the extent of EGFR phosphorylation on specific tyrosine residues by immunoblotting with phosphospecific monoclonal antibodies. IFN-γ treatment and *C. albicans* infection altered the autophosphorylation of specific EGFR tyrosine residues in two distinct patterns. The phosphorylation of Y992 and Y1045 increased progressively during *C. albicans* infection, but this increase was essentially unaffected by IFN-γ (Fig. 4B to D). In contrast, the phosphorylation of Y1068 increased, especially at 60 min postinfection, and this increase was blocked by IFN-γ (Fig. 4B and E).

SFKs phosphorylate EGFR on Y845 and Y1101, enhancing EGFR signaling (36). While *C. albicans* infection did not induce phosphorylation of either tyrosine residue, IFN-γ treatment significantly inhibited the phosphorylation of Y1101 (Fig. 4F to H). Collectively, these data suggest that the inhibitory effects of IFN-γ on the endocytosis of *C. albicans* are due to reduced phosphorylation of EGFR on Y1068 and/or Y1101. The finding that *C. albicans* and IFN-γ had the greatest effect on phosphorylation at the 60-min time point suggests that phosphorylation of these tyrosine residues may be required to prime the endocytosis signaling pathway.

**FIG 5:**
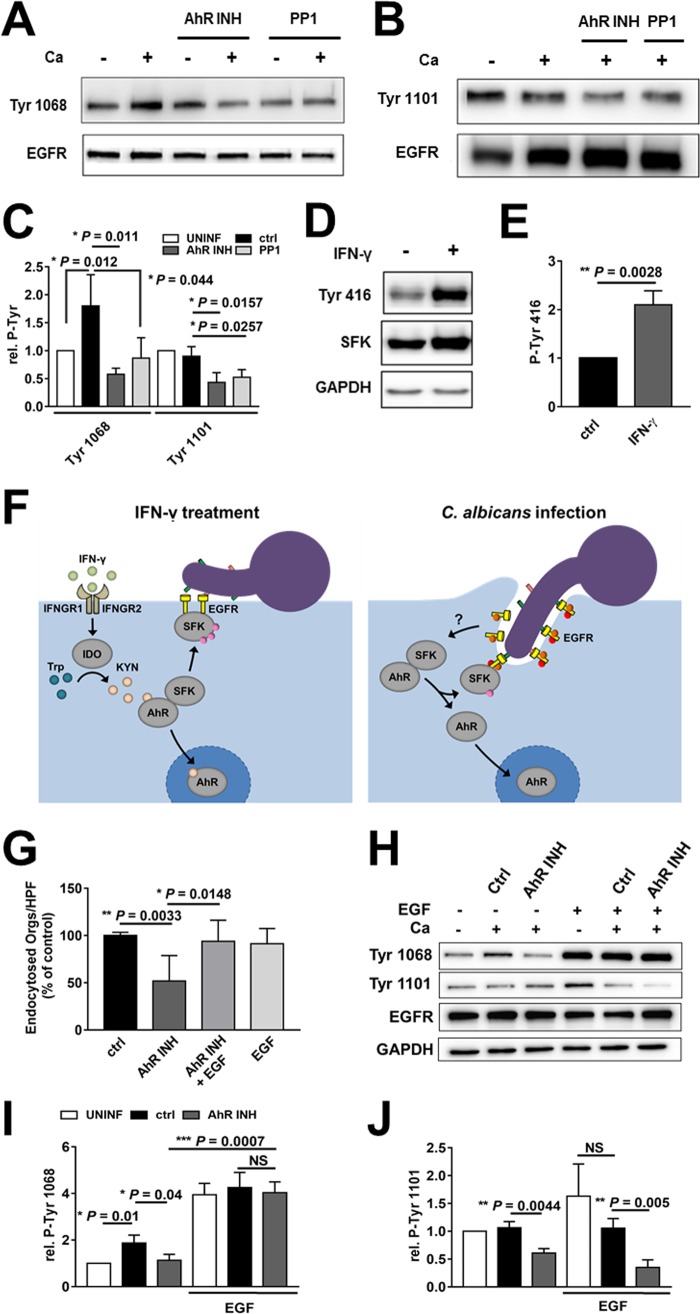
*C. albicans*-induced phosphorylation of EGFR depends on AhR and SFK activity. (A to C). Effects of inhibition of AhR and SFKs on *C. albicans*-induced phosphorylation of EGFR. OKF6/TERT-2 epithelial cells were pretreated for 1 h with the indicated inhibitor and then infected with *C. albicans* for 1 h. (A and B) Representative immunoblots showing EGFR phosphorylation at Y1068 (A) and Y1101 (B). (C) Densitometric analysis of the immunoblots in panels A and B. Results are means ± SD from 3 experiments. (D and E) Effects of IFN-γ on SFK phosphorylation. (D) Representative immunoblot. (E) Densitometric analysis of the immunoblots in panel D. Results are means ± SD from 3 experiments. (F, left panel) Proposed model of how IFN-γ inhibits the endocytosis of *C. albicans* by activating IDO, leading to the production of kynurenines that induce prolonged activation of AhR and SFKs, thereby preventing *C. albicans*-induced activation of EGFR and inhibiting endocytosis of the organism. (Right panel) Proposed model in which *C. albicans* activates AhR, stimulating SFKs that phosphorylate EGFR, leading to the endocytosis of the fungus. (G to J) Effects of the epidermal growth factor (EGF) and the AhR inhibitor on endocytosis (G) and EGFR phosphorylation (H to J). Results are means ± SD from three experiments, each performed in triplicate. Statistical significance was determined using the unpaired Student’s *t* test (*P* ≤ 0.05). Ca, *C. albicans*; INH, inhibitor; UNINF, uninfected; ctrl, control; Kyn, kynurenines; NS, not significant.

Next, we analyzed the effects of blocking AhR and SFKs on the phosphorylation of these tyrosine residues. Both the AhR and SFK inhibitors decreased *C. albicans*-induced phosphorylation of EGFR at Y1068 and Y1101 (Fig. 5A to C), similarly to what we observed with IFN-γ (Fig. 4). Incubation of epithelial cells with l-kynurenine for 24 h also inhibited phosphorylation of Y1068 and Y1101 (Fig. S5). Furthermore, incubating the epithelial cells with IFN-γ for 24 h stimulated the phosphorylation of SFKs (Fig. 5D and E), even though it inhibited phosphorylation of EGFR. These results suggest that prolonged stimulation of SFKs leads to compensatory downregulation of EGFR phosphorylation.

10.1128/mBio.00025-17.5 FIG S5  Effects of l-kynurenine on *C. albicans*-induced phosphorylation of EGFR. OKF6/TERT-2 epithelial cells were incubated with l-kynurenine for 24 h and then infected with *C. albicans* for 1 h (A) Representative immunoblots showing EGFR phosphorylation at Y1068 and Y1101. (B) Densitometric analysis of the immunoblots in panel A. Results are means ± SD from 3 experiments. Statistical significance was determined using the unpaired Student’s *t* test (*P* ≤ 0.05). Ca, *C. albicans*; UNINF, uninfected; L-Kyn, l-kynurinine. Download FIG S5, PDF file, 0.1 MB. Copyright © 2017 Solis et al. 2017 Solis et al. This content is distributed under the terms of the Creative Commons Attribution 4.0 International license.

Collectively, these data support a model in which prolonged exposure to IFN-γ upregulates epithelial cell IDO, stimulating the production of kynurenines and activating AhR and SFKs. Prolonged SFK activation inhibits phosphorylation of EGFR on Y1068 and Y1101 and blocks the endocytosis of *C. albicans* (Fig. 5F). Furthermore, by activating SFKs and inducing the phosphorylation of EGFR, AhR plays a key role in initiating the endocytosis of *C. albicans* by oral epithelial cells.

This model predicts that the effects of the AhR inhibitor could be reversed if EGFR remains phosphorylated. To test this prediction, we added epidermal growth factor (EGF), the natural ligand of EGFR, to oral epithelial cells that had been infected with *C. albicans* in the presence or absence of the AhR inhibitor. As predicted, EGF restored *C. albicans* endocytosis by epithelial cells treated with the AhR inhibitor but had no effect on endocytosis by untreated cells (Fig. 5G). We also analyzed the effects of EGF on the phosphorylation of EGFR at Y1068 and Y1101. EGF strongly stimulated the phosphorylation of Y1068, both in the presence and in the absence of *C. albicans*. This phosphorylation of was not reduced by the AhR inhibitor (Fig. 5H and I). In contrast, EGF did not enhance the phosphorylation of Y1101 in the presence of *C. albicans*, and the phosphorylation of this tyrosine residue was inhibited by the AhR inhibitor (Fig. 5H and J). These results suggest that AhR-induced phosphorylation of EGFR on Y1068, but not Y1101, is necessary for oral epithelial cells to endocytose *C. albicans*.

### IFN-γ, AhR, and SFKs have different effects on *C. albicans*-induced epithelial cell damage and cytokine release.

**FIG 6:**
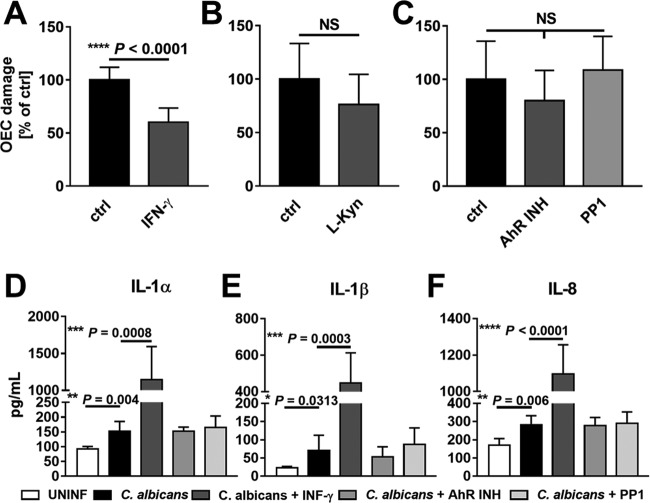
Effects of IFN-γ, AhR, and SFKs on epithelial cell damage and cytokine release induced by *C. albicans* (A to C). Oral epithelial cells were incubated with IFN-γ (A) or l-kynurenine (B) for 24 h or with the AhR or SFK inhibitor (C) for 1 h and then infected with *C. albicans* for 7 h. The extent of epithelial cell damage was measured using a ^51^Cr release assay. (D and E) Oral epithelial cells were incubated with the indicated compounds as in panels A to C and infected with *C. albicans* for 8 h, after which the supernatant was collected and analyzed for the concentration of interleukin-1α (IL-1α [D]), IL-1β (E), and IL-8 (F). Results are means ± SD from three experiments, each performed in triplicate. Statistical significance was determined using the unpaired Student’s *t* test (*P* ≤ 0.05). OEC, oral epithelial cells; ctrl, control; NS, not significant; INH, inhibitor; UNINF, uninfected; L-Kyn, l-kynurenine.

In addition to inducing its own endocytosis by epithelial cells, *C. albicans* damages these cells and stimulates them to produce proinflammatory cytokines (39). We investigated the effects of IFN-γ and the inhibition of AhR and SFKs on these responses. While treatment with IFN-γ significantly inhibited the extent of *C. albicans*-induced epithelial cell damage, treatment with l-kynurenine or the AhR or SFK inhibitor did not (Fig. 6A to C). Also, IFN-γ markedly enhanced the release of interleukin-1α (IL-1α), IL-1β, and IL-8 by epithelial cells in response to *C. albicans* infection (Fig. 6D to F). In contrast, neither the AhR nor the SFK inhibitor significantly altered the release of these cytokines by the infected epithelial cells. Collectively, these results indicate that while IFN-γ inhibits epithelial cell endocytosis of *C. albicans* by acting via IDO, AhR, and SFKs, it decreases epithelial cell damage and stimulates the release of proinflammatory cytokines via a different pathway or pathways.

### Inhibition of AhR ameliorates disease in the mouse model of OPC.

**FIG 7:**
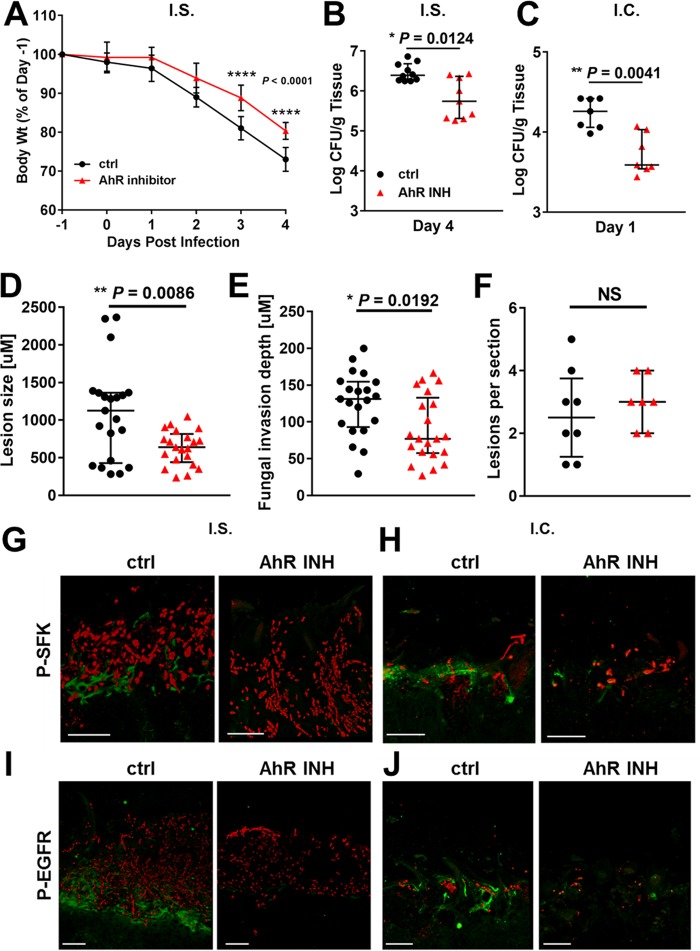
Inhibition of AhR reduces severity of disease during experimental OPC. Immunosuppressed (I.S.) and immunocompetent (I.C.) mice were treated with either diluent (control [ctrl]) or the AhR inhibitor (INH) and then orally inoculated with *C. albicans*. (A) Daily body weights of the immunosuppressed mice. (B and C) Oral fungal burden of the immunosuppressed mice after 4 days of infection (B) and of the immunocompetent mice after 1 day of infection (C). Results in panels A and B are medians ± interquartile ranges from the combined results of two separate experiments for a total of 9 to 10 mice per experimental group. Results in panel C are medians ± interquartile ranges from a single experiment with 7 mice per experimental group. (D to F) Analysis of the fungal lesions in tongues of immunosuppressed mice after 4 days of infection. (D) Length of the fungal lesions. (E) Depth of maximal fungal invasion. (F) Number of fungal lesions per tongue section. Results in panels D to F are medians ± interquartile ranges from the analysis of 7 to 8 thin sections from two separate experiments using a total of 6 mice per experimental group. (G to J) Inhibition of AhR reduces phosphorylation of SFKs and EGFR in the oral mucosa. Shown are confocal images of thin sections of the tongues of immunosuppressed (G and I) and immunocompetent (H and J) mice that were administered either diluent alone (ctrl [left panels]) or the AhR inhibitor (right panels) and then infected with *C. albicans* as in panels A to C. The thin sections in panels G and H were stained for phospho-SFK Y416 (green), and the thin sections in panels I and J were stained for phospho-EGFR Y1068 (green). All sections were also stained with an anti-*Candida* antiserum (red). Scale bar, 50 µm. Statistical significance was determined using the Mann-Whitney test (*P* ≤ 0.05).

To determine if AhR is required for the pathogenic interactions of *C. albicans* with epithelial cells *in vivo*, we analyzed the effects of the AhR inhibitor on disease severity in the mouse model of OPC. In mice that were immunosuppressed with cortisone acetate prior to induction of OPC, treatment with the AhR inhibitor limited the extent of weight loss and reduced the oral fungal burden by 8-fold relative to control mice that received the vehicle alone (Fig. 7A and B). In immunocompetent mice, treatment with the AhR inhibitor also significantly decreased the oral fungal burden (Fig. 7C). Quantitative analysis of thin sections of the tongues of the immunosuppressed mice demonstrated that the fungal lesions of mice treated with the AhR inhibitor were smaller and that the maximal depth of fungal invasion was shallower relative to the control mice (Fig. 7D and E). However, the numbers of fungal lesions were similar in both groups (Fig. 7F). Of note, the AhR inhibitor did not affect the length of the fungal hyphae, either *in vitro* or *in vivo* (see Fig. S6 in the supplemental material). In addition, the AhR inhibitor had no effect on the capacity of neutrophils to kill *C. albicans* (see Fig. S7 in the supplemental material)[Supplementary-material figS7]. Consistent with our *in vitro* data, oral infection with *C. albicans* induced phosphorylation of SFKs and EGFR in both immunosuppressed and immunocompetent mice (Fig. 7G to J). Moreover, treatment with the AhR inhibitor significantly inhibited this phosphorylation. These results indicate that signaling through AhR is necessary for *C. albicans* to activate SFKs and EGFR and to invade oral epithelial cells during the pathogenesis of OPC.

10.1128/mBio.00025-17.6 FIG S6  The AhR inhibitor has no effect on hyphal length. (A) Oral epithelial cells were incubated in the presence or absence of the AhR inhibitor for 1 h and infected with *C. albicans* for 2.5 h, after which the length of the fungal hyphae was determined. Results are means ± SD of 50 organisms. (B) Hyphal length of *C. albicans* in the tongues of mice with OPC after 4 days of infection. To detect *C. albicans* hyphae, thin sections of the infected tongues were rehydrated in PBS and then blocked. They were stained with an anti-*Candida* antibody conjugated with Alexa Fluor 568 and then imaged by confocal microscopy. The length of the elongated cells (>10 µm) was measured using LAS AF lite software. Results are means ± SD from 50 organisms in the tongues of 3 mice per experimental group. NS, not significant; ctrl, control. Download FIG S6, PDF file, 0.2 MB. Copyright © 2017 Solis et al. 2017 Solis et al. This content is distributed under the terms of the Creative Commons Attribution 4.0 International license.

10.1128/mBio.00025-17.7 FIG S7  Effects of the AhR inhibitor on the capacity of neutrophils to kill *C. albicans*. Human neutrophils were incubated with *C. albicans* cells at a ratio of 1:1 for 3 h in the presence of 10% pooled human serum, with or without the AhR inhibitor. The percentage of organisms killed was determined by colony counting. Results are means ± SD from 3 experiments. Statistical significance was determined using the unpaired Student’s *t* test (*P* ≤ 0.05). ctrl, control; INH, inhibitor. Download FIG S7, PDF file, 0.1 MB. Copyright © 2017 Solis et al. 2017 Solis et al. This content is distributed under the terms of the Creative Commons Attribution 4.0 International license.

## DISCUSSION

Invasion of oral epithelial cells is a vital step in the pathogenesis of OPC. By investigating the mechanism by which IFN-γ treatment protects oral epithelial cells from candidal invasion, we discovered that AhR plays a central role in governing EGFR-mediated endocytosis of *C. albicans* by oral epithelial cells, both *in vitro* and *in vivo*. This conclusion is supported by our findings that both prolonged activation of AhR by IFN-γ or l-kynurenine and inhibition of AhR by either siRNA or a small molecule inhibitor reduced the phosphorylation of EGFR and the endocytosis of *C. albicans in vitro*. Furthermore, treatment of both immunocompetent and immunocompromised mice with the AhR inhibitor ameliorated experimental OPC.

AhR is known to modulate the host inflammatory response to infectious agents via its effects on leukocytes. This receptor is required for maximal interleukin-10 (IL-10) production by NK cells (40). Also, by inhibiting NLRP3 expression in macrophages, AhR reduces the inflammatory response and inhibits apoptosis during infection (41, 42) In dendritic cells, AhR activates SFKs, which phosphorylate IDO, leading to increased enzyme activity and production of tolerogenic kynurenines (43). In the gastrointestinal mucosa, AhR induces IL-22 production by innate lymphoid cells, thereby augmenting the antifungal resistance of gastrointestinal epithelial cells (44). IL-22 is also necessary for the host’s defense against OPC (45); in contrast, the data presented here demonstrate that AhR has a proinfective function—it induces the endocytosis of *C. albicans* by acting through SFKs to stimulate the phosphorylation of EGFR, a key epithelial cell receptor for this organism.

Previously, we reported that when pregerminated *C. albicans* hyphae were added to oral epithelial cells, they stimulated EGFR phosphorylation within 10 min (21). The capacity of *C. albicans* hyphae to induce phosphorylation of EGFR so rapidly suggests that the fungus quickly stimulates AhR, leading to activation of one or more SFKs that phosphorylate EGFR. Because AhR is located in the cytoplasm, *C. albicans* must activate this receptor indirectly. Although kynurenines are one of the numerous endogenous AhR ligands, it seems unlikely that *C. albicans* stimulates IDO activity and induces sufficient synthesis of kynurenines to activate AhR within just 10 min. Thus, it is more probable that *C. albicans* stimulates AhR by inducing the release of a preformed endogenous ligand, the identity of which remains to be determined.

A notable finding was that although *C. albicans* infection stimulated the phosphorylation of multiple tyrosine residues in EGFR, the phosphorylation of only two of these residues, Y1068 and Y1101, was governed by IFN-γ. It has been reported that 15 min of exposure of A431, HeLa, and HEK-293 epithelial cells to IFN-γ induces phosphorylation of multiple tyrosine residues in EGFR, including Y1068, and that this phosphorylation can be blocked by inhibition of SFKs (46). In contrast, another group found that a 48-h treatment of human T84 colonic epithelial cells with IFN-γ downregulates the phosphorylation of Y1068 in response to EGF (47). Although the effects of IFN-γ on the phosphorylation of Y1101 were not tested by either of these groups, these data are consistent with our findings that IFN-γ activates SFKs and that prolonged exposure to IFN-γ induces a compensatory downregulation of EGFR phosphorylation.

Although *C. albicans* infection stimulated the autophosphorylation of multiple tyrosine residues of EGFR in oral epithelial cells, Y1068 appears to be the most important in governing the endocytosis of the fungus. Not only was Y1068 phosphorylated in response to *C. albicans* hyphae, but this phosphorylation was blocked by prolonged exposure to IFN-γ, l-kynurenine, and inhibition of either AhR or SFKs. Furthermore, treatment of epithelial cells with EGF reversed the inhibitory effect of the AhR inhibitor on Y1068 phosphorylation and restored endocytosis of *C. albicans*. Y1068 is known to bind to growth factor receptor binding protein 2 (Grb2) (48), an adapter protein that is required for EGFR to be internalized via clathrin-coated pits (49). Previously, we found that endocytosis of *C. albicans* is mediated by a clathrin-dependent mechanism (50). The present data suggest that *C. albicans* stimulates the phosphorylation of Y1068 of EGFR, which in turn activates the clathrin endocytosis pathway, leading to internalization of the fungus.

Although *C. albicans* did not induce the phosphorylation of EGFR Y1101, the basal level of phosphorylation of this tyrosine residue was reduced by IFN-γ, l-kynurenine, and the AhR and SFK inhibitors. However, when epithelial cells were treated with both EGF and the AhR inhibitor, they were able to endocytose *C. albicans*, even though phosphorylation of Y1101 was reduced. Thus, it is highly probable that Y1101 phosphorylation is dispensable for the induction of endocytosis.

Although treatment with IFN-γ and the AhR and SFK inhibitors had very similar effects on epithelial cell endocytosis of *C. albicans*, only IFN-γ inhibited fungus-induced epithelial cell damage and enhanced the release of proinflammatory cytokines. Previously, we had found that IFN-γ likewise protects endothelial cells from damage by *C. albicans* (27). The present data indicate that IFN-γ must protect oral epithelial cells from damage and augment cytokine release via signaling pathways that are independent of AhR and SFKs.

Cancer cell lines are a powerful tool dissecting the interactions of fungi with host cells. However, SFKs and EGFR are overexpressed in many epithelial cell lines (51, 52). The OKF6/TERT-2 oral epithelial cell line was developed by the forced expression of the human telomerase gene in oral keratinocytes from a healthy individual (28). Recently, we determined that the transcriptional response of OKF6/TERT-2 cells to *C. albicans* infection was highly similar to that of oral mucosa in mice with OPC. Specifically, *C. albicans* infection in OKF6/TERT-2 cells and OPC in mice activated the same signaling pathways, including the EGFR, IL-17, tumor necrosis factor (TNF), Toll-like receptor (TLR), and NF-κB pathways (53). In the present study, we found that AhR and SFKs are crucial for regulating EGFR signaling during the pathogenic interactions of *C. albicans* with OKF6/TERT-2 cells *in vitro* and during OPC in both immunosuppressed and immunocompetent mice. Thus, OKF6/TERT-2 cells constitute a powerful tool for elucidating the receptors and signaling pathways that govern the epithelial cell response to *C. albicans* during OPC.

Previously, we found that treatment of corticosteroid-treated mice with GW2974, an inhibitor of EGFR and HER2, blocked *C. albicans*-induced phosphorylation of these receptors and reduced the severity of OPC, demonstrating the importance of receptor-mediated fungal invasion of epithelial cells in the pathogenesis of this disease (21). In the present work, we determined that in corticosteroid-treated mice, a small molecule inhibitor of AhR markedly decreased the phosphorylation of SFKs and EGFR, and it ameliorated OPC similarly to GW2974. The AhR inhibitor was also efficacious in immunocompetent mice, although these animals clear *C. albicans* from the oral cavity very rapidly and do not exhibit overt OPC symptoms (45, 54). These results suggest that because AhR is essential for *C. albicans* to subvert EGFR signaling and invade epithelial cells *in vivo*, it is a potential therapeutic target.

## MATERIALS AND METHODS

### Ethics statement.

All animal work was approved by the Institutional Animal Care and Use Committee (IACUC) of the Los Angeles Biomedical Research Institute. The collection of blood from human volunteers for neutrophil isolation was also approved by the Institutional Review Board of the Los Angeles Biomedical Research Institute.

### Cells and cell lines.

*C. albicans* SC5314 (55) was used in all experiments. It was maintained on yeast extract-peptone dextrose agar (YPD). For use in the experiments, the organisms were grown for 18 h in YPD broth in a shaking incubator at 30°C. The next day, the fungal cells were harvested by centrifugation, washed twice with phosphate-buffered saline (PBS), and counted using a hemacytometer.

The human oral epithelial cell line OKF6/TERT-2 was kindly provided by J. Rheinwald (Harvard University, Cambridge, MA) (28) and was cultured as previously described (20). Recombinant IFN-γ (PeproTech) was reconstituted in Dulbecco’s PBS containing 0.1% bovine serum albumin (BSA) (Sigma) and stored in aliquots at −80°C. In all experiments, OKF6/TERT-2 cells were incubated with IFN-γ at a final concentration of 25 ng/ml for 24 h prior to infection with *C. albicans*, and the IFN-γ was present in the medium for the duration of the infection.

### Measurement of epithelial cell endocytosis.

The endocytosis of *C. albicans* by oral epithelial cells was quantified by a differential fluorescence assay as described previously (13). Briefly, OKF6/TERT-2 cells were grown to confluence on fibronectin-coated circular glass coverslips in 24-well tissue culture plates. They were infected with 2 × 10^5^ yeast-phase *C. albicans* cells per well and incubated for 2.5 h, after which they were fixed, stained, and mounted inverted on microscope slides. The coverslips were viewed with an epifluorescence microscope, and the number of endocytosed organisms per high-power field was determined, counting at least 100 organisms per coverslip. Each experiment was performed at least three times in triplicate.

To determine the effects of the antibodies, exogenous ligands, and inhibitors on endocytosis, the host cells were incubated with an anti-IFN-γ receptor monoclonal antibody (25 µg/ml; R&D Systems), 1-methyl-d-tryptophan (0.2 mM; Sigma-Aldrich), l-kynurenine (100 µM; Sigma-Aldrich), levo-1-methyl tryptophan (l-1MT) (0.2 mM; Sigma-Aldrich), 3,4-DAA (200 µM; Cayman Chemical), CH-223191 (10 µM; Sigma-Aldrich), gefitinib (1 µM; Selleckchem), PP1 (100 nM; Cell Signaling), KX2-391 (100 nM; Selleckchem), or EGF (50 ng/ml; Life Technologies, Inc.). The inhibitors were added to the host cells 60 min before infection with *C. albicans*, and they remained in the medium for the entire incubation period. Control cells were incubated with a similar concentration of the diluent (dimethyl sulfoxide [DMSO]) at final concentrations ranging from 0.1 to 0.2%.

As described previously (21), siRNA was used to deplete AhR from the epithelial cells. OKF6/TERT-2 cells were transfected with random control siRNA (Qiagen) or AhR siRNA (80 pmol; Santa Cruz Biotechnology) using Lipofectamine 2000 (Thermo Fisher Scientific) following the manufacturer’s instructions.

### RNA-seq and real-time PCR.

For RNA-seq, OKF6/TERT-2 cells in six-well tissue culture plates were treated with either recombinant IFN-γ or medium alone for 24 h and then infected with 1 × 10^7^ *C. albicans* yeast cells for 5 h in biological triplicates. Total epithelial cell RNA was isolated using the RiboPure yeast kit (Ambion), according to the manufacturer’s instructions. The RNA was subjected to poly(A) enrichment by the TruSeq protocol, after which RNA-seq libraries (non-strand-specific, paired end) were prepared with the TruSeq RNA kit (Illumina). Using the HiSeq platform, 100 nucleotides of sequence was determined from each end of the cDNA fragments. Sequencing reads were aligned to the human reference genome Ensemble GRCh38 (56) using TopHat2 (57). The alignment files were then used to generate read counts for each gene, and a statistical analysis of differential gene expression was assessed using the DESeq package from Bioconductor (58). Reads per kilobase million (RPKM) values for each gene in each sample were generated using in-house scripts. For real-time PCR, host RNA was extracted using the RiboPure yeast kit, according to the manufacturer’s instructions. After preparing cDNA, the transcript levels of the genes of interest were measured by real-time PCR using the primers listed in Table S3 in the supplemental material. The relative transcript level of each gene was normalized to GAPDH (glyceraldehyde-3-phosphate dehydrogenase) by the threshold cycle (2^−ΔΔ*CT*^) method.

10.1128/mBio.00025-17.10 TABLE S3  Oligonucleotides used in the experiments. Download TABLE S3, DOCX file, 0.1 MB. Copyright © 2017 Solis et al. 2017 Solis et al. This content is distributed under the terms of the Creative Commons Attribution 4.0 International license.

### Kynurenine measurement.

OKF6/TERT-2 cells in 24-well tissue culture plates were incubated with medium alone, IFN-γ, l-1MT, or IFN-γ plus l-1MT. After 24 h, the medium above the cells was collected, clarified by centrifugation, and stored at −80°C. The amount of l-kynurenine in the conditioned medium was determined by enzyme-linked immunosorbent assay (ELISA) (MyBioSource) according to the manufacturer’s instructions.

### Detection of protein phosphorylation.

OKF6/TERT-2 cells in six-well tissue culture plates were incubated in tissue culture medium with or without IFN-γ for 24 h and then infected with 4.5 × 10^6^ *C. albicans* cells. At various time points, the cells were rinsed with cold PBS containing protease and phosphatase inhibitor cocktails and removed from the plate with a cell scraper. The cells were collected by centrifugation and boiled in sample buffer. The lysates were separated by SDS-PAGE, and the phosphorylation of specific tyrosine residues of EGFR was detected by immunoblotting with specific antibodies (phospho-EGF receptor antibody sampler kit 9922 from Cell Signaling and EGFR–p-Tyr1101 from ECM Biosciences). Next, the blot was stripped, and total EGFR was detected by immunoblotting with an anti-EGFR antibody (catalog no. sc-101; Santa Cruz Biotechnology). Following a similar approach, SFK phosphorylation on Y416 was determined using the antibodies in the Src antibody sampler kit 9935 (Cell Signaling). Each experiment was performed at least 3 times.

### Indirect immunofluorescence.

To determine the intracellular location of AhR, OKF6/TERT-2 cells were incubated in tissue culture medium with or without IFN-γ or l-kynurenine for 24 h. Next, the cells were fixed with 3% paraformaldehyde, blocked with 10% BSA, and incubated with an anti-AhR antibody (catalog no. sc-133088; Santa Cruz Biotechnology), followed by an Alexa 488-conjugated mouse anti-rabbit antibody. To visualize the nuclei, the cells were also stained with DAPI (4′,6-diamidino-2-phenylindole). The cells were then imaged by confocal microscopy. To visualize the perimeters of the epithelial cells, they were also imaged by differential interference contrast.

### Flow cytometry.

The expression of EGFR, HER2, and E-cadherin on the surface of the oral epithelial cells was quantified by flow cytometry. Briefly, OKF6/TERT-2 cells in 6-well tissue culture plates were incubated with tissue culture medium with or without IFN-γ for 24 h and then infected with 5 × 10^5^ *C. albicans* cells. After 75 min, the cells were scraped from the wells with a cell scraper, fixed with 3% paraformaldehyde, blocked with 1% goat serum, and then stained with specific antibodies (for EGFR, sc-101, and for HER2, sc-33684, from Santa Cruz Biotechnology; for E-cadherin, ab1416 from Abcam, Inc.), followed by an Alexa 488-conjugated goat or mouse anti-rabbit antibody (Life Technologies, Inc.). Control epithelial cells were incubated in a similar concentration or mouse or rabbit IgG (Abcam, Inc.). The fluorescence of the cells was determined by flow cytometry, analyzing at least 10,000 cells per condition.

### Host cell damage assay.

The extent of oral epithelial cell damage caused by the different treatments was measured using our previously described ^51^Cr release assay (22). Briefly, OKF6/TERT-2 cells were grown to 95% confluence in 96-well tissue culture plates with detachable wells (Corning) and loaded with 5 µCi/ml Na_2_^51^CrO_4_ (PerkinElmer) in the presence or absence of IFN-γ or l-kynurenine for 24 h. After removing the unincorporated ^51^Cr by rinsing, the epithelial cells were infected with 6 × 10^5^ *C. albicans* cells. When the AhR and SFK inhibitors were used, they were added to the cells 60 min before infection with *C. albicans*, and they remained in the medium for the entire incubation period. After 7 h, the amount of ^51^Cr released into the medium and retained by the cells was determined by gamma counting. Each experiment was performed three times in triplicate.

### Cytokine production.

To measure the release of cytokines, OKF6/TERT-2 cells in a 96-well tissue culture plate were incubated with IFN-γ for 24 h or the AhR and SFK inhibitors for 60 min prior to infection. Next, 3 × 10^5^ yeast-phase *C. albicans* cells were added to the cells. After 8 h, the supernatant was collected, clarified by centrifugation, and stored at −80°C. The concentrations of IL-8/CXCL8, IL-1α, and IL-1β in the medium were determined using the Luminex multiplex assay (R&D Systems). Each experiment was performed three times in triplicate.

### Mouse model of oropharyngeal candidiasis.

The effect of AhR inhibitor on the severity of OPC was determined in both immunocompromised and immunocompetent mice following our standard protocol (59). Male BALB/c mice were fed an oral solution of the AhR inhibitor (10 mg/kg/day), administered in divided doses twice daily in 0.05 ml of a 1:1 mixture of propylene glycol and water starting on day −1 relative to infection. Control mice received an equal volume of the vehicle alone. When immunocompromised mice were used, cortisone acetate (2.25 mg/kg) was administered subcutaneously on days −1, 1, and 3 (59). For inoculation, the animals were sedated with ketamine and xylazine, and a swab saturated with 10^6^ *C. albicans* cells was placed sublingually for 75 min. Immunocompetent mice were inoculated similarly, except that the swab was saturated with 2 × 10^7^ organisms. The immunocompromised and immunocompetent mice were sacrificed after 4 days and 1 day of infection, respectively. Next the tongue and attached tissues were harvested and divided longitudinally. One hemisection was weighed, homogenized, and quantitatively cultured, and the other was processed for histology.

To detect phosphorylation of EGFR, and SFKs, 2-μm-thick sections of OCT-embedded tongues were fixed with cold acetone. Next, the cryosections were rehydrated in PBS and then blocked. They were stained with EGFR–p-Tyr1068 (Cell Signaling) and P-Src-Tyr416 (Cell Signaling) primary antibodies and then rinsed and stained with an Alexa Fluor 488 secondary antibody. To detect *C. albicans*, the sections were also stained with an anti-*Candida* antiserum (Biodesign International) conjugated with Alexa Fluor 568 (Thermo Fisher Scientific). The sections were imaged by confocal microscopy. To enable comparison of fluorescence intensities among slides, the same image acquisition settings were used for each experiment.

For histopathologic analysis, thin sections of paraffin-embedded tongues were stained with periodic acid-Schiff stain (PAS). The sections were imaged by light microscopy, and the length of the individual fungal lesions and the depth of fungal invasion relative to surface of the tongue were determined using Infinity Analysis software (Lumenera).

### Neutrophil killing.

The effects of the AhR inhibitor on neutrophil killing of *C. albicans* were determined as described elsewhere (60). Briefly, neutrophils were isolated from the blood of healthy volunteers and incubated with the AhR inhibitor in RPMI 1640 medium plus 10% fetal bovine serum for 1 h at 37°C. Next, the neutrophils were mixed with an equal number of *C. albicans* cells. After a 3-h incubation, the neutrophils were lysed by sonication, and the number of viable *C. albicans* cells was determined by quantitative culture.

### Statistics.

Data were compared by Mann-Whitney or unpaired Student’s *t* test using GraphPad Prism (v. 6) software. *P* values of <0.05 were considered statistical significant.

### Accession number(s).

All of the raw sequencing reads have been submitted to the NCBI Sequence Read Archive (SRA; https://www.ncbi.nlm.nih.gov/sra) under ID code SRP077728, BioSample numbers SAMN05150838, SAMN05150839, SAMN05150840, SAMN06392618, SAMN06392619, and SAMN06392620.
