# Supplementary material for: The Aryl Hydrocarbon Receptor Governs Epithelial Cell Invasion during Oropharyngeal Candidiasis
Source: mBio. 2017 Mar 21;8(2):e00025-17. doi: 10.1128/mBio.00025-17 (PMC5362030; doi:10.1128/mBio.00025-17)
Supplement: FIG S1 [file mbo002173240sf1.pdf]

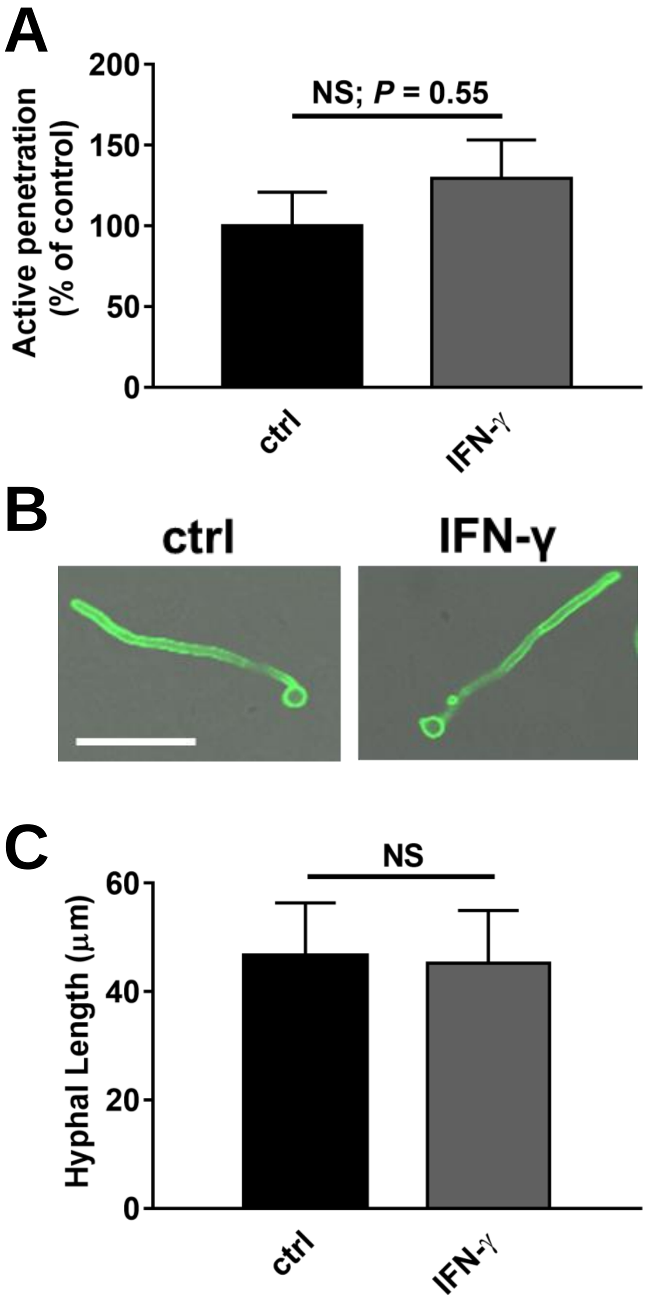

Figure S1 IFN-γ has no effect on active epithelial cells penetration or hyphal growth. (A) OKF6/TERT-2 oral epithelial cells were incubated with IFN-γ for 24 h, fixed, and infected for 2.5 h with *C. albicans* cells in the presence of IFN-γ, after which the number of internalized organisms was determined by a differential fluorescence assay. Results are mean ± SD of 3 experiments, each performed in triplicate. (B) Microscopic images of *C. albicans* cells after a 2.5 h incubation with oral epithelial cells that had been exposed to the indicated conditions for 24 h. The fungal cells where stained with an anti-*Candida* antiserum conjugated with Alexa Fluor488. (C) Hyphal length after incubation for 2.5 h on oral epithelial cells that had been exposed to the indicated conditions for 24 h. Results are mean ± SD of 50 organisms. Statistical significance was determined using the unpaired Student's t-test ($P \leq 0.05$).
