# Supplementary material for: The Aryl Hydrocarbon Receptor Governs Epithelial Cell Invasion during Oropharyngeal Candidiasis
Source: mBio. 2017 Mar 21;8(2):e00025-17. doi: 10.1128/mBio.00025-17 (PMC5362030; doi:10.1128/mBio.00025-17)
Supplement: FIG S2 [file mbo002173240sf2.pdf]

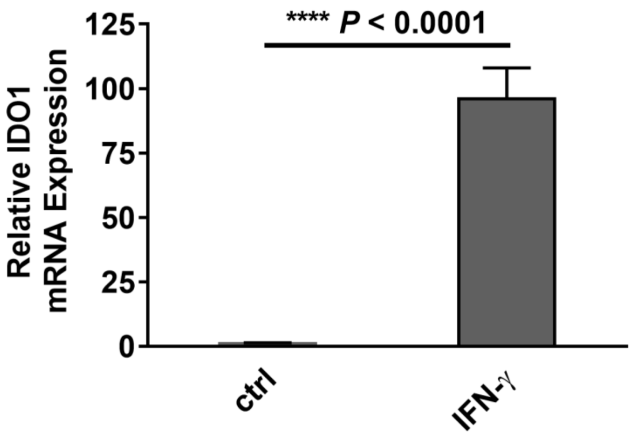

Figure S2 Effect of IFN-γ on epithelial cell IDO1 mRNA expression, as measured by real-time PCR. Results are mean ± SD of 2 experiments, each performed in triplicate. Statistical significance was determined using the unpaired Student's t-test ($P \leq 0.05$).
