# Supplementary material for: The Aryl Hydrocarbon Receptor Governs Epithelial Cell Invasion during Oropharyngeal Candidiasis
Source: mBio. 2017 Mar 21;8(2):e00025-17. doi: 10.1128/mBio.00025-17 (PMC5362030; doi:10.1128/mBio.00025-17)
Supplement: FIG S3 [file mbo002173240sf3.pdf]

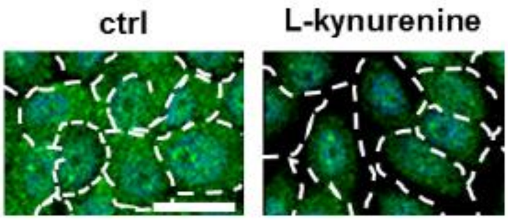

Figure S3 L-kynurenine activates the aryl hydrocarbon receptor (AhR) in oral epithelial cells. Confocal micrographs of OKF6/TERT-2 oral epithelial cells incubated in the presence and absence of L-kynurenine for 24 h. The cells were stained for AhR (green) and the nuclei were stained with DAPI (blue). The perimeters of the cells were determined by differential interference contrast and are indicated by the dashed lines. Scale bar 20 μm
