# Supplementary material for: The Aryl Hydrocarbon Receptor Governs Epithelial Cell Invasion during Oropharyngeal Candidiasis
Source: mBio. 2017 Mar 21;8(2):e00025-17. doi: 10.1128/mBio.00025-17 (PMC5362030; doi:10.1128/mBio.00025-17)
Supplement: FIG S4 [file mbo002173240sf4.pdf]

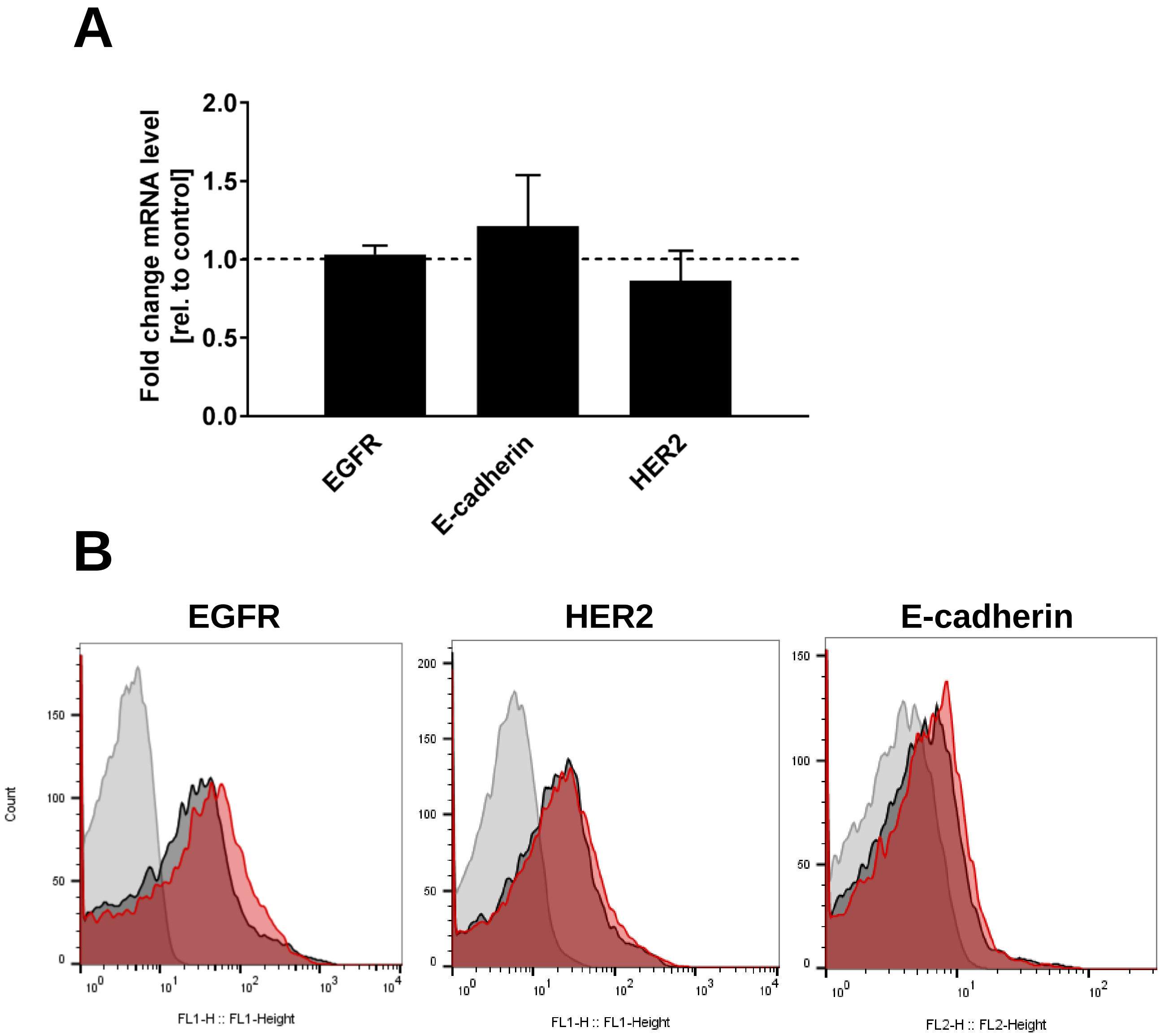

Figure S4 IFN-γ treatment has no effect on the expression of host cell receptors for *C. albicans* (A) mRNA levels of the indicated epithelial cell receptors after 24 h IFN-γ treatment. Results are the mean ± SD of two independent experiments performed in triplicate. (B) Effects of IFN-γ on the expression of E-cadherin, EGFR and HER2 on the surface of epithelial cells as determined by flow cytometry. Control cells (stained with a control mAb) are shown in light gray, untreated cells (stained with the specific mAb) are shown in dark grey, and the IFN-γ treated cells (stained with the specific mAb) are shown in red.
