# Supplementary material for: The Aryl Hydrocarbon Receptor Governs Epithelial Cell Invasion during Oropharyngeal Candidiasis
Source: mBio. 2017 Mar 21;8(2):e00025-17. doi: 10.1128/mBio.00025-17 (PMC5362030; doi:10.1128/mBio.00025-17)
Supplement: FIG S5 [file mbo002173240sf5.pdf]

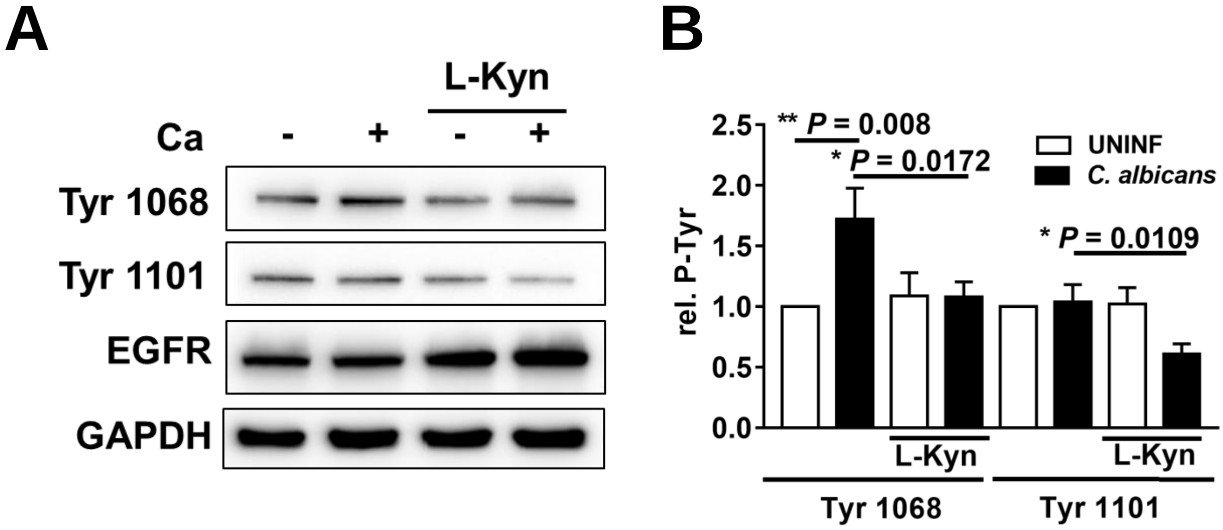

Figure S5 Effects of L-kynurenine on *C. albicans* induced phosphorylation of EGFR. OKF6/TERT-2 epithelial cells were incubated with L-kynurenine for 24 h and then infected with *C. albicans* for 1 h. (A) Representative immunoblots showing EGFR phosphorylation at Y1068 and Y1101. (B) Densitometric analysis of the immunoblots in (A). Results are the mean ± SD of 3 experiments. Statistical significance was determined using the unpaired Student's t-test ($P \leq 0.05$).
