# Supplementary material for: The Aryl Hydrocarbon Receptor Governs Epithelial Cell Invasion during Oropharyngeal Candidiasis
Source: mBio. 2017 Mar 21;8(2):e00025-17. doi: 10.1128/mBio.00025-17 (PMC5362030; doi:10.1128/mBio.00025-17)
Supplement: FIG S6 [file mbo002173240sf6.pdf]

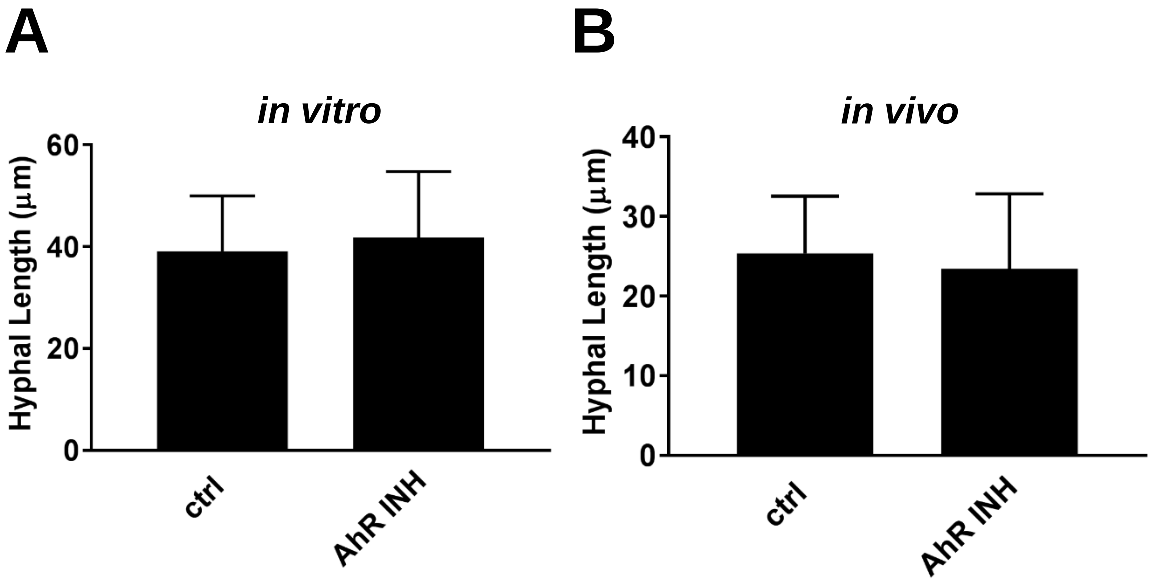

Figure S6 The AhR inhibitor has no effect on hyphal length. (A) Oral epithelial cells were incubated in the presence or absence of the AhR inhibitor for 1 h and infected with *C. albicans* for 2.5 h, after which the length of the fungal hyphae was determined. Results are the mean ± SD of 50 organisms. (B) Hyphal length of *C. albicans* in the tongues of mice with OPC after 4d of infection. To detect *C. albicans* hyphae, thin sections of the infected tongues were rehydrated in PBS and then blocked. They were stained with an anti-*Candida* antibody conjugated with AlexaFluor 568 and then imaged by confocal microscopy. The length of the elongated cells (>10 µm) was measured using LAS AF lite software. Results are the mean ± SD of 50 organisms in the tongues of 3 mice per experimental group.
