# Supplementary material for: The Aryl Hydrocarbon Receptor Governs Epithelial Cell Invasion during Oropharyngeal Candidiasis
Source: mBio. 2017 Mar 21;8(2):e00025-17. doi: 10.1128/mBio.00025-17 (PMC5362030; doi:10.1128/mBio.00025-17)
Supplement: FIG S7 [file mbo002173240sf7.pdf]

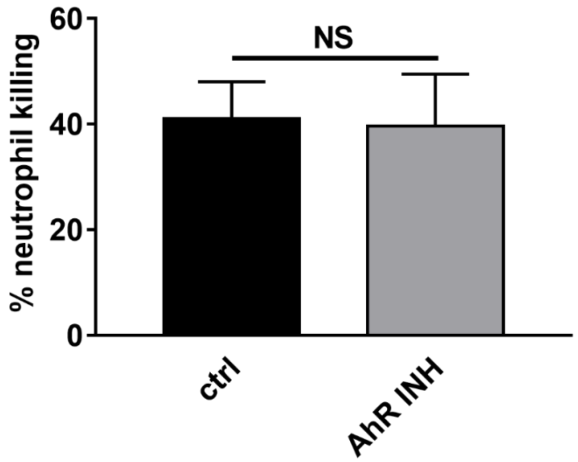

Figure S7 Effects of the AhR inhibitor on the capacity of neutrophils to kill *C. albicans.* Human neutrophils were incubated with *C. albicans* cells at a ratio of 1:1 for 3 h in the presence of 10% pooled human serum, with or without the AhR inhibitor. The percentage of organisms killed was determined by colony counting. Results are the mean ± SD of 3 experiments. Statistical significance was determined using the unpaired Student's t-test ($P \leq 0.05$).
