# Supplementary figures and images for: The Aryl Hydrocarbon Receptor Governs Epithelial Cell Invasion during Oropharyngeal Candidiasis
Source: mBio. 2017 Mar 21;8(2):e00025-17. doi: 10.1128/mBio.00025-17 (PMC5362030; doi:10.1128/mBio.00025-17)

**
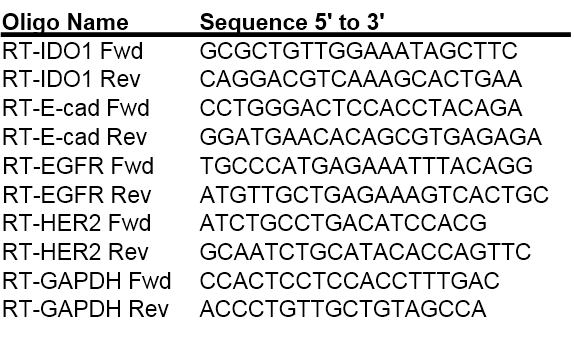
Table S3**. Oligonucleotides used in the experiments

Supplement: TABLE S3 [file mbo002173240st3.docx]
